# Blue carbon in human-dominated estuarine and shallow coastal systems

**DOI:** 10.1007/s13280-015-0725-x

**Published:** 2015-11-03

**Authors:** Tomohiro Kuwae, Jota Kanda, Atsushi Kubo, Fumiyuki Nakajima, Hiroshi Ogawa, Akio Sohma, Masahiro Suzumura

**Affiliations:** Coastal and Estuarine Environment Research Group, Port and Airport Research Institute, 3-1-1, Nagase, Yokosuka, 239-0826 Japan; Department of Ocean Sciences, Tokyo University of Marine Science and Technology, 4-5-7 Konan, Minato-ku, Tokyo, 108-8477 Japan; Department of Urban Engineering, The University of Tokyo, 7-3-1 Hongo, Bunkyo-ku, Tokyo, 113-8656 Japan; Atmosphere and Ocean Research Institute, The University of Tokyo, 5-1-5, Kashiwanoha, Kashiwa-shi, Chiba, 277-8564 Japan; Mizuho Information and Research Institute, 2-3, Kanda-Nishikicho, Chiyoda-ku, Tokyo, 101-8443 Japan; National Institute of Advanced Industrial Science and Technology, AIST Tsukuba West, 16-1 Onogawa, Tsukuba, 305-8569 Japan

**Keywords:** Carbon cycles, Carbon sequestration, Climate change, CO_2_ fluxes, Urban ecology

## Abstract

Estuarine and shallow coastal systems (ESCS) are recognized as not only significant organic carbon reservoirs but also emitters of CO_2_ to the atmosphere through air–sea CO_2_ gas exchange, thus posing a dilemma on ESCS’s role in climate change mitigation measures. However, some studies have shown that coastal waters take up atmospheric CO_2_ (C_atm_), although the magnitude and determinants remain unclear. We argue that the phenomenon of net uptake of C_atm_ by ESCS is not unusual under a given set of terrestrial inputs and geophysical conditions. We assessed the key properties of systems that show the net C_atm_ uptake and found that they are often characteristic of human-dominated systems: (1) input of high terrestrial nutrients, (2) input of treated wastewater in which labile carbon is highly removed, and (3) presence of hypoxia. We propose that human-dominated ESCS are worthy of investigation as a contributor to climate change mitigation.

## Introduction

Quantifying carbon stocks, identifying and locating atmospheric CO_2_ (C_atm_) sinks and sources, and understanding their drivers are important for climate change mitigation. Carbon transported from land and taken up from the atmosphere is stored within the ocean in various forms of organic and inorganic matter (IPCC 2013). In particular, coastal ecosystems are recognized as significant carbon reservoirs because of their high carbon burial rates and long-term sequestration of organic carbon (McLeod et al. 2011; Fourqurean et al. 2012; Duarte et al. 2013). So-called “blue carbon,” termed by UNEP in 2009, is the carbon captured by marine living organisms (Nellemann et al. 2009). Estimates of stored blue carbon in a variety of shallow coastal ecosystems such as mangrove forests, salt marshes, seagrass meadows, and intertidal flats are ongoing (e.g., Chmura et al. 2003; Donato et al. 2011; Breithaupt et al. 2012; Fourqurean et al. 2012; Duarte et al. 2013; Miyajima et al. 2015). The organic carbon stored in shallow coastal sediment and within organisms is critical as a source of CO_2_ if significantly disturbed and oxidized by anthropogenic impacts, such as physical destruction and deterioration of water and sediment quality (McLeod et al. 2011; Fourqurean et al. 2012).

Despite growing understanding of the significance of blue carbon, there have been few syntheses of the efficacy of estuarine and shallow coastal systems (ESCS) as a means of climate change mitigation (Nellemann et al. 2009; McLeod et al. 2011; Duarte et al. 2013). Prediction is challenging because the dynamics of such waters are particularly complex; the presence of intertwined interfaces (air–water, water–sediment, air–sediment, and freshwater–saltwater) is associated with diverse biogeochemical cycles and biota as well as high exchange rates between interfaces. Nevertheless, there are some studies dealing with the stocks and fluxes of both organic and inorganic carbon in ESCS (Maher and Eyre 2012; Obrador and Pretus 2012; Tokoro et al. 2014) and the estimates are largely unconstrained (Cai 2011; Chen et al. 2013; Laruelle et al. 2013; Regnier et al. 2013).

A controversial point, less debated but still critical from the standpoint of blue carbon climate change mitigation, is that ESCS are generally recognized to be net emitters of CO_2_ to the atmosphere through air–sea CO_2_ gas exchange (e.g., Borges et al. 2005; Cai 2011; Chen et al. 2013; Laruelle et al. 2013; Regnier et al. 2013); although some studies have shown C_atm_ uptake (e.g., Kone et al. 2009). Hence, their role in climate change mitigation is paradoxical: ESCS indeed sequester carbon derived from the atmosphere yet they also emit CO_2_ to the atmosphere. Further, due to the geographical location of ESCS and the socio-economic history of use, the carbon fluxes of ESCS have long been altered by human activities (Bauer et al. 2013; Regnier et al. 2013). In particular, because wastewater treatment has a major impact on human-dominated coastal areas (McIntyre et al. 2000), we hypothesize that wastewater treatment affects C_atm_ exchange and benthic carbon storage.

Here, we discuss carbon stocks and fluxes related to climate change mitigation in ESCS. We targeted systems with a salinity of 1–33, the rationale being the definition of estuaries as areas affected by both freshwater and saltwater. Therefore, we excluded continental shelves. By conceptualizing recent findings, we argue that the phenomenon of net uptake of C_atm_ by ESCS is not unusual. We summarize the key factors that determine whether ESCS exhibit net uptake. Further, we argue that the relevance of anthropogenic impacts to air–sea CO_2_ fluxes and carbon burial in ESCS will become increasingly important to the study of human system-ecosystem interactions. Also, we suggest the need for further investigation of several overlooked coastal processes that potentially contribute to carbon sequestration from the atmosphere.

## Dilemma of blue carbon in climate change mitigation measures

Here, we discuss the dilemma of ESCS functioning related to climate change mitigation; both positive as a carbon reservoir and negative as a net emitter of CO_2_ to the atmosphere. However, we note that functioning as both a carbon reservoir and a net emitters of CO_2_ to the atmosphere is scientifically compatible (not paradoxical), as carbon flow in such ecosystems includes carbon transported from land, which is partly stored within ESCS, partly mineralized and outgassed, with the rest outflowed to the open ocean.

Recently, ESCS have been identified as areas of substantial blue carbon storage, particularly in the sediment (Nellemann et al. 2009; McLeod et al. 2011; Fourqurean et al. 2012; Duarte et al. 2013). Of the biogeochemical factors that influence carbon sequestration in sediments (e.g., Canfield 1994; Hartnett et al. 1998; Zonneveld et al. 2010; Koho et al. 2013), large amounts of allochthonous and autochthonous organic matter (Kennedy et al. 2010), mineral particles from rivers, and the mixing and settling (flocculation) of these materials may be responsible for high burial rates of carbon in ESCS (Sholkovitz 1976). As sediments vertically accrete, carbon burial occurs continuously unless significant, long-term elevation changes occur (e.g., sediment loss due to erosion and subsidence). The carbon that accumulates in sediments mineralizes slowly in subsurface layers where anoxic conditions exist and can be isolated from the earth’s atmosphere for millennia (Chambers et al. 2001).

Shallow, vegetated coastal waters have the highest carbon burial rates in the ocean (average: 138–226 g C m^−2^ year^−1^, range: 18–1713 g C m^−2^ year^−1^), at least three orders of magnitude higher than in open ocean sediments (0.018 g C m^−2^ year^−1^) (Nellemann et al. 2009; McLeod et al. 2011). The difference is not fully explained by the difference in net ecosystem production between shallow vegetated coastal waters (1044–2784 g C m^−2^ year^−1^) and the open ocean (120 g C m^−2^ year^−1^) (Gattuso et al. 1998). In shallow vegetated systems, restriction of water movement by vegetation stimulates trapping of particulate organic matter (POM) and carbon burial (Hendriks et al. 2007; Kennedy et al. 2010).

However, as noted, ESCS have been regarded as emitters of CO_2_, a consequence of the input of terrestrial carbon and its subsequent mineralization and respiration. Regnier et al. (2013) reported that the land–ocean aquatic continuum (comprised of freshwaters, estuaries, and continental shelves) is both a net emitter of CO_2_ (0.35 Pg C year^−1^) and a net carbon storage (0.55 ± 0.28 Pg C year^−1^). Such an apparent contradiction is the basis of the controversy about the role of ESCS in climate change mitigation measures.

## Requirements for a long-term net uptake of C_atm_

Unlike sequestration in terrestrial ecosystems, aquatic carbon burial is not directly linked with the removal of C_atm_, because the water column, within which complex inorganic and organic biogeochemical processes occur, separates the atmosphere from benthic systems. Only when the partial pressure of CO_2_ (pCO_2_) of the water is lower than that of the atmosphere at the air–water interface will a system absorb C_atm_ (Wanninkhof 1992). Given that fluctuations of atmospheric pCO_2_ levels are small compared with fluctuations of surface water pCO_2_, factors that regulate surface water pCO_2_ are primarily responsible for determining the direction of the flux.

Lowering of surface water pCO_2_ is facilitated if allochthonous carbon (inorganic and organic) inputs are low and there are mechanisms to consume CO_2_ and suppress CO_2_ production. However, river water generally has a high pCO_2_ (Raymond et al. 2013; Regnier et al. 2013); the pCO_2_ values of 95 % of global inland waters are higher than atmospheric pCO_2_, the median pCO_2_ being 3100 μatm (Raymond et al. 2013). Thus, for ESCS surface waters to become undersaturated with respect to pCO_2_, some process within the system must decrease the pCO_2_ of the water. Such processes include a decrease of temperature and dissolved inorganic carbon (DIC) as well as an increase of total alkalinity (TA).

**Fig. 1:**
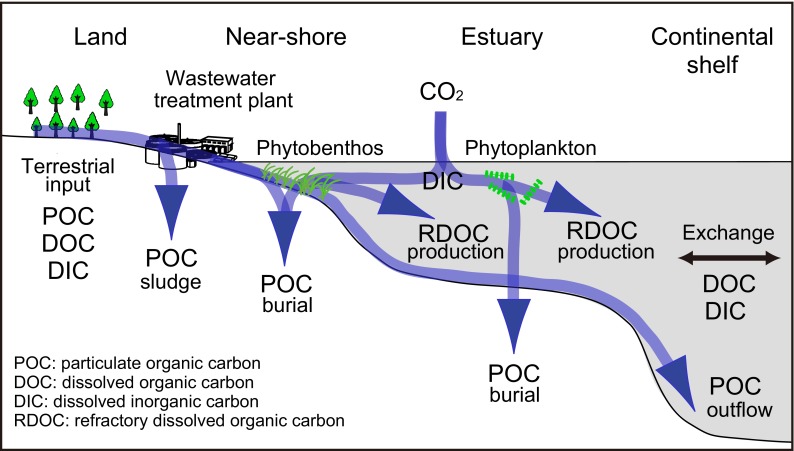
Conceptualized carbon flows that contribute to a long-term net uptake of C_atm_ in ESCS. The conceptual diagram is based on the assumption that a long-term net uptake (at least over years) of C_atm_ occurs only when there is a net unidirectional carbon flux (pump) that leads to a partial pressure of CO_2_ (pCO_2_) in water lower than the atmospheric pCO_2_. Lowering of pCO_2_ is enhanced by (1) primary production by phytoplankton and phytobenthos (submerged aquatic vegetation), (2) suppression of mineralization and release of CO_2_ by production of refractory dissolved organic matter (RDOC), (3) sedimentation and burial of particulate organic matter (POC), and (4) efflux of POC into the deep sea. Wastewater treatment plants can indirectly contribute to lowering of pCO_2_ in water by removal of POC (sludge) in terrestrial inputs; however, the plants can also be emitters of CO_2_ to the atmosphere due to water treatment (oxygenation of wastewater and mineralization of organic matter in open treatment basins)

For a system to show a long-term net uptake of C_atm_, there must be a net unidirectional carbon influx (pump) when fluxes are averaged over years (Fig. 1). Of the various types of relevant carbon fluxes, burial of organic carbon in sediments, production of refractory dissolved organic carbon (RDOC), and export of particulate organic carbon (POC) are particularly important for the pump. All three of these processes have geological turnover rates and contribute to the suppression of mineralization and the resultant return of CO_2_ to the atmosphere.

## Mechanistic hypothesis for long-term C_atm_ uptake in human-dominated escs

**Fig. 2:**
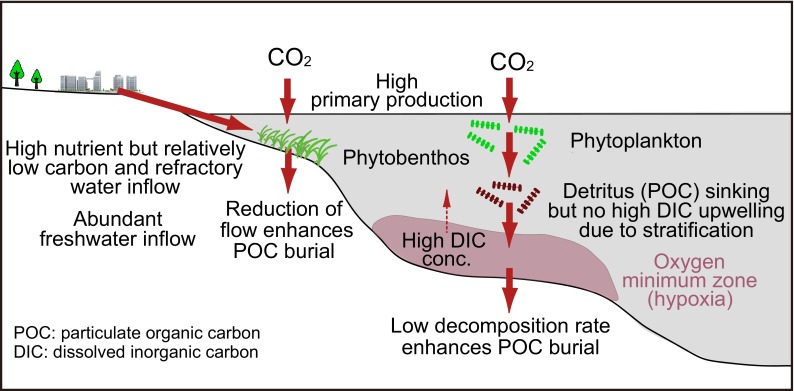
Key processes and mechanisms that explain why human-dominated ESCS show net uptake of C_atm_ at least over years through air–sea CO_2_ gas exchange. For details, see Table 1

Human impacts on ESCS alter carbon cycling. One can expect that these impacts alter C_atm_ exchanges and benthic carbon storage by increasing nutrient loading, wastewater treatment, and freshwater use (McIntyre et al. 2000). Below, by synthesizing mechanistic hypotheses and empirical evidence from previous studies (Table 1), we conceptualize that human impacts are closely related to the creation of C_atm_ uptake. Our hypothesis is that discharges of high-nutrient but relatively low-carbon water generated by wastewater treatment, as well as an increase in freshwater discharges due to importation of freshwater and watershed alteration, are key to enhancing direct uptake of C_atm_ in ESCS (Fig. 2). We acknowledge that our hypothesis should be quantitatively validated by numerical modeling.

**Table 1:** Key processes and mechanisms relevant to air–sea CO_2_ fluxes and carbon burial in human-dominated ESCS

Property	Driver	Consequence	Relevance to atmospheric CO_2_ uptake and carbon burial
Large amount of nutrient input	Nutrient input from land	Enhancement of high primary production	Low pCO_2_ in water
Relatively small amount of labile carbon input	Wastewater treatment (removal and mineralization of organic carbon)	Relatively low-carbon mass input to sea Suppression of mineralization but less suppression of primary production	Low pCO_2_ in water
Large amount of freshwater discharge	Freshwater demand due to population (importation of water, watershed alteration)	Enhancement of stratification Suppression of upwelling of high-DIC-concentration bottom waters due to stratification Low turbidity in surface water due to suppression of resuspension and upwelling of POC from bottom water, enhancing light availability and photosynthesis	Low pCO_2_ in surface water
Presence of oxygen minimum zone (OMZ)	Stratification High organic matter input	Anoxia/hypoxia in both bottom water and surface sediments Suppression of mineralization Production of POC by anoxic/hypoxic polymerization	Enhancement of carbon burial
Shallow water depth	Geological settings	Short degradation time during POC sinking in water column	Enhancement of carbon burial
Turbidity	Plankton blooming Mineral particle input from terrestrial	Enhancement of primary production due to increase in phytoplankton biomass, lowering pCO_2_ Suspended particles suppressing light availability and photosynthesis, raising pCO_2_	Variability of pCO_2_ in surface water
Residence time	Freshwater input Water exchange at the boundary	Influenced by the quantity and quality (pCO_2_ and POC) of inflowing water	Variability of pCO_2_ in surface water and carbon burial

### Wastewater treatment

Wastewater treatment has a considerable impact on biogeochemical cycles in human-dominated ESCS (Grimm et al. 2008; Kaushal and Belt 2012; Kubo et al. 2015). We propose that wastewater treatment can contribute to the creation of the long-term net uptake of C_atm_ in human-dominated ESCS for two reasons. First, current wastewater treatment, such as the conventional activated sludge method, removes carbon in the form of sludge and CO_2_ gas more efficiently than nutrients such as nitrogen and phosphorus (Sedlak 1991). Through these effluents, the balance of primary production and respiration in the ESCS is offset towards an excess of primary production and the resultant suppression of any pCO_2_ increase. Second, the effluent contains relatively refractory carbon, because labile organic matter has already been removed by treatment (Kubo et al. 2015). Therefore, respiration and mineralization rates of effluent are low, and subsequent pCO_2_ increases are suppressed.

### Freshwater use, stratification, and hypoxia

Human populations rely on large amounts of freshwater, and watershed hydrologic alteration is often employed to meet demands (Grimm et al. 2008; Kaushal and Belt 2012). An increase of runoff and possibly the temperature by human use enhances stratification and water exchanges in ESCS, thereby indirectly but significantly affecting biogeochemical cycles in these systems.

Stratified water hinders upwelling of high-DIC bottom water and suppresses increases of DIC and pCO_2_ in the surface water and air–sea CO_2_ efflux. However, unlike the effect of stratification on DIC behavior, sinking of POM from the surface water is not hindered by stratification (Kone et al. 2009). Thus, POM, which is the potential source of DIC in surface waters, moves downward via sinking, independent of the degree of stratification (Fig. 2). In particular, because ESCS have shallow water columns, the time for settling of POM to the bottom is shorter (little POM is mineralized) than in the case of the open ocean, where most POM is mineralized before reaching the bottom. Also, stratification suppresses resuspension from bottom waters as well as upwelling of resuspended particles (turbidity). Therefore, transparency is enhanced, light is more available for photosynthesis, and the pCO_2_ of the surface water is lowered (Chen et al. 2008).

Stratification is seasonal in temperate and boreal regions, occurring mainly in summer. Such seasonality may, in turn, influence the dynamics of air–sea CO_2_ exchanges. During summer, stratification blocks upwelling of bottom waters containing high concentrations of DIC. During other seasons, in the absence of stratification, high-DIC bottom water is mixed with surface water, the result being an increase of pCO_2_ in surface water and enhancement of the CO_2_ efflux into the atmosphere. However, lower temperatures during these times suppress the efflux of CO_2_ because of the negative correlation between CO_2_ solubility and temperature.

Mineralization rates are acknowledged to be low under low-oxygen conditions (Hartnett et al. 1998; Koho et al. 2013), although the issue is contentious (Canfield 1994). Because lability related to redox potential is dependent on the nature of the organic matter (Zonneveld et al. 2010), we expect that less organic matter is mineralized in a narrower range of redox potential than in a broader range (e.g., having both negative and positive redox potential variations). Thus, the hypoxia or oxygen minimum zone (OMZ) that forms in a stratified water column can geochemically enhance carbon burial because redox ranges in the sediment will be narrower than without OMZ. Further, biological processes also mediate carbon burial in the OMZ. For example, Koho et al. (2013) reported that carbon burial is high in the OMZ because macrofaunal manipulation of organic carbon particles and bioturbation are less prevalent under hypoxic conditions. However, the OMZ also enhances production of potent greenhouse gases (N_2_O and CH_4_), offsetting climate change mitigation.

Turbidity affects surface water pCO_2_ both positively and negatively. If the source of turbidity mainly consists of phytoplankton, primary production can be enhanced by an increase in phytoplankton biomass, leading to lowering of pCO_2_. In turn, if the source mainly consists of mineral particles, light availability and photosynthesis are suppressed, leading to raising of pCO_2_ (Chen et al. 2012). Also, residence time is a critical factor for determining both surface water pCO_2_ and carbon burial, affecting both positively and negatively because they are influenced by the quantity and quality (pCO_2_ and POC) of inflowing water (Gazeau et al. 2005).

### Field studies

Here we show published data for ESCS influenced in key ways by anthropogenic impacts. Since estimated global average CO_2_ fluxes for small deltas, tidal systems, and lagoons, net emitters of CO_2_ to the atmosphere, have already been compiled elsewhere (Laruelle et al. 2013), we focused on previous field cases where ESCS showed C_atm_ uptake (Table 2) using available databases (Google Scholar and Scopus). In most instances, we found that the ESCS showing C_atm_ uptake were, variously, impacted by treated wastewater, stratified, or characterized by the presence of hypoxia. Inputs of wastewater, clearly related to land use impacted by anthropogenic activities (urban and farmland), led to high concentrations of nutrients and chlorophyll *a*, and high primary production rates in the ESCS. Positive relationships between net ecosystem production (metabolism) and C_atm_ absorption have been shown (Maher and Eyre 2012; Tokoro et al. 2014). In shallow coastal ecosystems, we found that air–sea CO_2_ fluxes were negative (ESCS takes up C_atm_) only where seagrass meadows were present, although there are reports showing undersaturated pCO_2_ [e.g., kelp beds (Delille et al. 2009) and coral reefs (Kayanne et al. 1995, 2005)]. The result may, in part, reflect that in this study, we focused on ecosystems where the salinity was 1–33, which is uncommonly low for kelp beds and coral reefs. We found that the magnitude of the average CO_2_ influx was higher in seagrass meadows (24.6 ± 44.1 mmol C m^−2^ day^−1^, mean ± SD) than in estuarine systems (9.6 ± 6.7 mmol C m^−2^ day^−1^, mean ± SD). In contrast, estimated global average CO_2_ fluxes were positive (effluxes) for small deltas (40.3 mmol C m^−2^ day^−1^), tidal systems (41.4 mmol C m^−2^ day^−1^), and lagoons (49.9 mmol C m^−2^ day^−1^) (Laruelle et al. 2013).

**Table 2:** Environmental conditions when the air–sea flux is negative (atmospheric CO_2_ uptake) in estuarine and shallow coastal ecosystems (ESCS) (salinity range: 1–33). Only the references showing the flux values are listed. Note that most of the summarized data here were measured as snapshots (not 24-h continuous measurements) and did not include an annual cycle and associated with considerable uncertainties and are possibly biased. For comparison, estimated global average CO_2_ fluxes are positive (emitters): 40.3 mmol C m^−2^ day^−1^ for small deltas, 41.4 mmol C m^−2^ day^−1^ for tidal systems, and 49.9 mmol C m^−2^ day^−1^ for lagoons (Laruelle et al. 2013)

Location	Site condition				Surface water condition					Flux and measurement condition			Reference
	Land use	Treated waste water input	Stratification	Oxygen minimum zone	Temp. (°C)	Salinity	DIN or NO_3_ (μM)	DIP (μM)	Chl*-a* (μg L^−1^)	Air–sea negative CO_2_ flux (mmol C m^−2^ day^−1^)	Measurement intervals	Measurement season	
Estuarine systems													
Noordwijk	Urban/farmland	Yes	Yes	–	15–17	29–32	–	–	–	<20.0	24 h continuous	September	Bakker et al. (1996)
York River estuary	Urban/forest	Yes	Yes	–	–	14–24	–	–	–	2.1–5.6	Daytime snapshot	November–April	Raymond et al. (2000)
Randers Fjord	Farmland	Yes	Yes	No	9–10	5–12	55–90	0.2–0.3	2–6	10.0	24 h	April	Gazeau et al. (2005)
Tendo Lagoon	Farmland	No	Yes	Yes	31	1–7	1	0.3	24	17.7	Snapshot	March	Kone et al. (2009), Kouame et al. (2009)
Tendo Lagoon	Farmland	No	Yes	Yes	–	0–3	1	0.3	27	4.9	Snapshot	September	Kone et al. (2009), Kouame et al. (2009)
Tendo Lagoon	Farmland	No	Yes	Yes	–	0–2	2	0.6	8	3.0	Snapshot	December	Kone et al. (2009), Kouame et al. (2009)
Aby Lagoon	Farmland	No	Yes	Yes	30	5–12	0	0.3	28	20.0	Snapshot	March	Kone et al. (2009), Kouame et al. (2009)
Aby Lagoon	Farmland	No	Yes	Yes	–	1–10	1	0.2	36	11.3	Snapshot	September	Kone et al. (2009), Kouame et al. (2009)
Aby Lagoon	Farmland	No	Yes	Yes	–	2–7	2	0.2	17	4.1	Snapshot	December	Kone et al. (2009), Kouame et al. (2009)
Aby Lagoon	Farmland	No	Yes	Yes	–	–	–	–	–	7.4	Snapshot	Annual average	Kone et al. (2009), Kouame et al. (2009)
Bellamy River estuary	Urban/farmland	No	–	–	9–12	10–20	9	0.3	3–7	12.0	–	April	Hunt et al. (2011)
Oyster River estuary	Urban/farmland	Yes	–	–	10–11	4–18	18	0.4	4–5	17.2	–	April	Hunt et al. (2011)
Neuse River estuary	Urban/farmland	Yes	Yes	–	16–31	2–15	–	–	5–20	0.8	Daytime continuous	June–August	Crosswell et al. (2012)
Neuse River estuary	Urban/farmland	Yes	Yes	–	8–9	4–7	–	–	12–20	22.0	Daytime continuous	December–February	Crosswell et al. (2012)
Neuse River estuary	Urban/farmland	Yes	Yes	–	8	2–4	–	–	12–27	2.4	Daytime continuous	March–May	Crosswell et al. (2012)
Neuse River estuary	Urban/farmland	Yes	Yes	–	8–29	4–19	–	–	7–20	0.5	Daytime continuous	Annual average	Crosswell et al. (2012)
Godthabsfjord	Icecap	–	–	–	–1–7	24–34	–	0–1	–	20.0	Snapshot	Annual average	Rysgaard et al. (2012)
Columbia River estuary	Urban/farmland	Yes	Yes	Yes	10	3–14	40	1	6	6.5–9.5	Daytime continuous	April	Evans et al. (2013)
Osaka Bay	Urban	Yes	Yes	Yes	7–28	16–32	7–21	–	10–50	8.3	Snapshot	Annual average	Fujii et al. (2013)
Tokyo Bay	Urban	Yes	Yes	Yes	8–32	2–35	0–374	0–32	0–300	8.8	Daytime snapshot	Annual average	Kubo (2015)
Simple mean and SD										9.6 ± 6.7			
Seagrass meadows, %cover													
Hasting River 10 %	Farmland	Yes	–	–	–	>10	–	–	–	1.0	Day/night	Annual average	Maher and Eyre (2012)
Camden Haven 37 %	Forest	Yes	–	–	–	>0	–	–	–	5.0	Day/night	Annual average	Maher and Eyre (2012)
Wallis Lake 37 %	Forest	Yes	–	–	–	>0	–	–	–	5.0	Day/night	Annual average	Maher and Eyre (2012)
Albufera des Grau	Urban/farmland	Yes	Yes (slight)	Yes	–	5–15	–	–	0–200	8.1	Daytime snapshot	Annual average	Obrador and Pretus (2012)
Shiraho	Farmland	No	No	No	29–33	32–34	–	–	–	1.9	24 h	September	Watanabe et al. (2013)
Furen Lagoon 80 %	Farmland	Yes	Yes	No	20–27	7–22	1–121	0.3–2.6	1–7	6.0–10.4	24 h continuous	August	Tokoro et al. (2014)
Furen Lagoon 80 %	Farmland	Yes	Yes	No	16–30	4–22	1–121	0.3–2.6	<18	126.0	24 h continuous	July–August	Tokoro et al. (2014)
Furen Lagoon 80 %	Farmland	Yes	Yes	No	11–27	6–31	1–121	0.3–2.6	–	1.5	Daytime snapshot	Annual average	Tokoro et al. (2014)
Kurihama Bay	Urban	Yes	No	No	12–14	29–33	8–29	0.4–1	1–4	2.5	Daytime snapshot	March and April	Tokoro et al. (2014)
Fukido Estuary	Forest	No	No	No	29–33	29–34	1–2	<0.1	<2	86.4	24 h continuous	August	Tokoro et al. (2014)
Simple mean and SD										24.6 ± 44.1			

However, we note that most summarized data were measured as snapshots (not 24-h continuous measurements) and did not include an annual cycle. The implication is that these data and average fluxes are associated with considerable uncertainties and are possibly biased. In particular, diurnal pCO_2_ changes are significant in low-salinity environments, where the carbonate buffer effect is weak, but few studies have documented the changes. Hence, we could not use statistical models to analyze the flux data and infer the drivers.

## Unexplored but important processes

Of the anticipated carbon fluxes that can contribute to removal of C_atm_, an important pathway is related to RDOC (Ogawa et al. 2001; Jiao et al. 2014; Kubo et al. 2015). Of the riverine carbon flux to the global coastal ocean, the DOC flux (246 Tg C year^−1^) comprises 28 % of the total carbon flux (869 C year^−1^) (Cai 2011). Air–sea CO_2_ fluxes in ESCS can be affected by how much DOC is mineralized into CO_2_ by microbial and photochemical processes (Moran et al. 2000) and how much new RDOC is produced within the systems (Fig. 1). The new production of RDOC within these systems is a largely unexplored process. RDOC is reported to be derived from phytoplankton (Kragh and Søndergaard 2009), bacteria (Ogawa et al. 2001; Lønborg et al. 2009), phytobenthos (Wada et al. 2008), and corals (Tanaka et al. 2011a, b). As above, inputs of organic matter with rich nutrients and less labile organic carbon can lower water column pCO_2_ and enhance C_atm_ uptake in ESCS; however, these conditions may decrease RDOC concentrations due to the priming effect (remobilization of RDOC by bacterial use of nutrients) (Taylor and Townsend 2010; Jiao et al. 2014). Thus, we note that the impact of interactions between DOC and nutrients on carbon sequestration is complex. Further, to evaluate the role of DOC in carbon sequestration, we need to quantify how much new DOC is synthesized from both allochthonous and autochthonous sources. However, identification and quantification of the source of new DOC remains technologically challenging because of the low concentration of DOC and the presence of salt, which interferes with quantification.

Carbon taken up by vegetation, such as seagrasses and seaweeds, and exported and sequestered in the deep sea, where it is mineralized and returned to the atmosphere as CO_2_ on a geological time-scale, is an important mechanism for C_atm_ sequestration (Kennedy et al. 2010) (Fig. 1). The extent of burial or degradation of seagrass shoots and rhizomes is largely unknown, but the magnitude of the global export flux has been estimated to be 0.05–0.1 Pg C year^−1^ (McLeod et al. 2011). In addition, new production (or transformation by polymerization) of RPOC within ESCS (both in the water column and sediment) (Zonneveld et al. 2010) is a process that may contribute to C_atm_ sequestration. However, this process has yet to be investigated.

## Future directions

### Need for more air–sea CO_2_ flux data

Compared to terrestrial and open ocean ecosystems, available data for CO_2_ gas exchanges and associated parameters in ESCS are scarce (Laruelle et al. 2013) and have yet to be included in the assessment report of the IPCC. A suite of key data for carbon cycling, such as air–water CO_2_ fluxes, carbonate chemistry, and organic carbon dynamics in coastal areas, are necessary to ascertain whether the various ESCS show net uptake or emission of CO_2_ (Maher and Eyre 2012; Obrador and Pretus 2012; Tokoro et al. 2014). Further, long-term air–sea CO_2_ flux data are needed to assess the variability and uncertainty of the flux associated with timescales, which is important for consideration of gas exchange-based accounting of CO_2_ as well as possible future anthropogenic impacts on air–sea CO_2_ exchange. Indeed, a numerical simulation has predicted that the trend of air–sea CO_2_ exchange is towards an increasing net uptake in coastal waters (including continental shelves) due to rising atmospheric pCO_2_ and increasing inorganic nutrient loads (Andersson and Mackenzie 2004).

#### Re-evaluation of sequestered carbon

Most studies related to blue carbon in ESCS and the importance of conserving these systems hinge on the assumption that if such ecosystems were lost, all the stored carbon would be mineralized and released into the atmosphere as CO_2_ (e.g., McLeod et al. 2011; Fourqurean et al. 2012). However, such an assumption is a worst-case scenario and further investigation is required (Pendleton et al. 2012; Macreadie et al. 2014). Finally, there is the argument over whether or not buried carbon derived from outside ESCS should be included with the autochthonous as carbon sequestered by ESCS. Some reports have estimated the separate proportion of autochthonous and allochthonous carbon contributions (Middelburg et al. 1997; Kennedy et al. 2010; Dubois et al. 2012; Watanabe and Kuwae 2015). The same argument would be true for whether or not exported POC should be included as carbon sequestered by ESCS.

### Mitigation by management of wastewater treatment

Carbon removal in wastewater treatment means that a significant part of the carbon removed is released as CO_2_ from the treatment plant instead of from the ESCS water surface. That is, if we view the process of CO_2_ gas exchange as part of an integrated system involving the entire coasts (i.e., the area including both the ESCS and the land where treatment plants are built), then C_atm_ uptake by the ESCS may be canceled out by CO_2_ emissions from the treatment plants. However, by appropriate management of the treatment process, we can benefit from both reduction of carbon emissions and atmospheric carbon uptake. For example, generated sludge can be utilized as a biofuel and generated CO_2_ for in situ capture and storage, and we can choose anaerobic treatments (e.g., methane fermentation) to use the emitted gas as a biofuel (Parkin and Owen 1986). In addition, we can manage the efficiency of carbon and nutrient removals as well as the quality of the treated water by selecting alternative treatment methods (e.g., coagulating sedimentation method, anaerobic–anoxic–oxic method). These human system–ecosystem interactions are complex, and biogeochemical modeling and numerical simulations appear necessary to inform optimal management of ESCS-based measures for mitigation and adaptation to climate change.

## Conclusion

ESCS are recognized as not only significant carbon reservoirs but also emitters of CO_2_ to the atmosphere through air–sea CO_2_ gas exchange, posing the dilemma of how ESCS functions relate to climate change mitigation measures. By synthesizing mechanistic hypotheses and empirical evidence from previous studies, we argue that the capability of ESCS to function as direct net uptake of C_atm_ over years is not exceptional and CO_2_ uptake is closely related to environmental conditions typical of human-dominated systems. Thus, our study offers a new perspective on the potential of human-dominated ESCS as a contributor to climate change mitigation, i.e., both carbon reservoirs and direct net uptake of C_atm_, in light of human systems–ecosystem interactions. In particular, investigation should be focused on vegetated ecosystems in ESCS, e.g., mangrove forests, salt marshes, and seagrass meadows as these ecosystems have high carbon burial rates and long-term carbon storage capability. Further, ecosystem conservation, restoration, and management appear more feasible to implement compared to other climate change mitigation options, such as ocean fertilization and geological carbon storage (Nellemann et al. 2009; Cusack et al. 2014). However, validating the potential of ESCS will require field studies to allow development of improved hydrographic-biogeochemical ESCS models.
